# Evaluation of synthetic vascular grafts in a mouse carotid grafting model

**DOI:** 10.1371/journal.pone.0174773

**Published:** 2017-03-29

**Authors:** Alex H. P. Chan, Richard P. Tan, Praveesuda L. Michael, Bob S. L. Lee, Laura Z. Vanags, Martin K. C. Ng, Christina A. Bursill, Steven G. Wise

**Affiliations:** 1 The Heart Research Institute, Sydney, New South Wales, Australia; 2 Sydney Medical School, University of Sydney, Sydney, New South Wales, Australia; 3 Royal Prince Alfred Hospital, Sydney, New South Wales, Australia; 4 School of Life and Environmental Sciences, University of Sydney, Sydney, New South Wales, Australia; Albany Medical College, UNITED STATES

## Abstract

Current animal models for the evaluation of synthetic grafts are lacking many of the molecular tools and transgenic studies available to other branches of biology. A mouse model of vascular grafting would allow for the study of molecular mechanisms of graft failure, including in the context of clinically relevant disease states. In this study, we comprehensively characterise a sutureless grafting model which facilitates the evaluation of synthetic grafts in the mouse carotid artery. Using conduits electrospun from polycaprolactone (PCL) we show the gradual development of a significant neointima within 28 days, found to be greatest at the anastomoses. Histological analysis showed temporal increases in smooth muscle cell and collagen content within the neointima, demonstrating its maturation. Endothelialisation of the PCL grafts, assessed by scanning electron microscopy (SEM) analysis and CD31 staining, was near complete within 28 days, together replicating two critical aspects of graft performance. To further demonstrate the potential of this mouse model, we used longitudinal non-invasive tracking of bone-marrow mononuclear cells from a transgenic mouse strain with a dual reporter construct encoding both luciferase and green fluorescent protein (GFP). This enabled characterisation of mononuclear cell homing and engraftment to PCL using bioluminescence imaging and histological staining over time (7, 14 and 28 days). We observed peak luminescence at 7 days post-graft implantation that persisted until sacrifice at 28 days. Collectively, we have established and characterised a high-throughput model of grafting that allows for the evaluation of key clinical drivers of graft performance.

## Introduction

Coronary artery bypass grafting is a common treatment for revascularisation of ischemic heart disease, especially in the case of diffuse multi-vessel disease. Autologous conduits such as the internal mammary artery (IMA) and saphenous vein are most commonly used, with the IMA in particular giving exceptional long-term clinical results [1]. However, a large portion of patients do not have sufficient autologous conduits available due to disease or prior use, meaning bilateral internal mammary grafting is rare, despite it’s proven superiority in treating multi-vessel disease [2]. Synthetic options such as ePTFE and Dacron, which are successful in large diameter applications, uniformly fail in low diameter (<6 mm) applications such as in the coronaries and the peripheries [3]. Significant and ongoing research in regenerative medicine and tissue engineering aims to develop new synthetic conduits, effective for low diameter revascularisation to meet this need.

Innovation in engineering new conduits relies heavily on animal models for pre-clinical assessment. An ideal animal model should reflect the human physiological response to vascular grafting, with a focus on the dominant modes of graft failure: neointimal hyperplasia (NH) and poor endothelialisation [4]. In addition, appropriate animal models should be cost effective, readily accessible to researchers, and deliver useful endpoints within a reasonable time frame. Comprehensive review of the literature suggests that the established pre-clinical pathway for graft evaluation in medium to large animal models should include rabbit and sheep carotid grafting [5]. However, studies involving medium to large animals are expensive and require significant expertise and equipment, making them difficult to access for basic research labs. Small animals are regularly employed as a model of mid-term immune response and graft remodelling, the most common being the rat abdominal aortic replacement. This model requires long-term time points for NH development and endothelialisation (at least 3 months). Also absent from this evaluation are established small animal models which facilitate ready simulation of disease and transgenic strains to study the mechanisms of graft failure and the vascular biology underpinning these processes. A mouse model of vascular grafting which reproducibly manifests the key drivers of graft failure would be immensely valuable.

Previous mouse grafting models have focused on replacing segments of the abdominal aorta using end to end suture methods. This approach has been used to evaluate a number of graft materials including decellularised ovine coronary artery, polyurethane and ePTFE [6]. However, the surgery is technically challenging due to the small diameter of the vessel and the need for multiple suture points required in a short time frame. It also requires the mobilisation of the abdominal cavity, which taken together with the location of the abdominal aorta, limit its compatibility with some non-invasive imaging techniques. Sutureless mouse grafting models utilising cuff anastomoses have previously been used in the context of vein and aortic transplants in both the carotid and abdominal aorta position [7, 8]. The carotid lies much closer to the surface making it easier to expose surgically and eliminating the need to temporarily remove visceral organs. The proximity to the surface also facilitates non-invasive imaging, including bioluminescent imaging. The utility of this sutureless method was previously shown using a vein graft in apolipoprotein E knockout (ApoE^-/-^) mice, following treatment with a chemokine inhibitor [9]. However, evaluation of synthetic vascular grafts using this method has yet to be characterised.

In this study, we comprehensively characterise a sutureless interposition grafting model which facilitates the evaluation of synthetic grafts in the mouse carotid artery. Using conduits electrospun from poly-caprolactone (PCL) we show the gradual development of a significant neointima and near-complete endothelialisation within 28 days, together replicating two critical aspects of graft performance. Furthermore, using longitudinal non-invasive tracking we characterise the homing and engraftment of luminescent/fluorescent bone-marrow mononuclear cells to the PCL graft.

## Material and methods

### Electrospinning

Polyaprolactone (M.W. 80 kDa, Sigma-Aldrich) was dissolved in 1,1,1,3,3,3-hexafluro-2-propanol (HFP) (Sigma-Aldrich) to a final concentration of 10% (w/v). Polymer solutions were electrospun onto a stainless steel mandrel (0.5 mm diameter) rotating at 500 rpm, at a rate of 2 mL/hr from a 20G flat needle, 20 kV, and an air gap distance of 20 cm. Grafts were sterilised in ethanol (70%) for 30 mins at room temperature followed by three washes in sterile PBS.

### Mouse carotid grafting

All procedures were approved by the Sydney Local Heath District Animal Welfare Committee, protocol number 2015/016A. Experiments were conducted in accordance with the Australian Code of Practice for the Care and Use of Animals for Scientific Purpose. All personnel involved in the animal procedures have completed an approved animal care and ethics course.

C57/BL6 mice (male, 9–10 weeks old, 25 ± 2 g) were purchased from Animal Resources Centre (Canning Vale, WA, Australia). One week before the surgery, the drinking water of mice were supplemented with aspirin (10 mg/kg/d). The cuff procedure was carried out as previously described [10] to implant synthetic graft materials into the carotid position. Briefly, the mice were anaesthetised with methoxyflurane (2%) and placed in a supine position. The right common carotid artery was mobilised and double ligated at the midpoint then dissected. Polyimide cuffs (Cole-Parmer) were placed around each end of the arteries and clamped. Over hanging arteries were everted on the plastic cuff and secured with 8–0 silk sutures to form the anastomoses. PCL grafts were sleeved over each end and secured with 8–0 sutures. Clamps were removed and blood flow was confirmed with pulsation. The skin was closed with 6–0 silk sutures and mice allowed to recover with food and water ad libitum. Mice received analgesic in the form of a single dose of carprofen (5 mg/kg) immediately post-surgery. The mice were monitored daily for five consecutive days post-surgery for signs of discomfort and administered additional analgesic if necessary. Mice that sustained a weight loss of 15% of the pre-surgery weight or had markedly reduce mobility were sacrificed by cervical dislocation.

Recipient mice were sacrificed at 7, 14 and 28 days after implant. The mice were anaesthetised with methoxyflurane (2%) and sacrificed by cardiac puncture. Finally, mice were perfused with heparinised saline and the grafted carotid artery was isolated and dissected proximal and distal to the graft.

The survival rate of the procedure was 83% (40 of 48), and procedure time 40±5 mins. Cause of death of the 8 mice were due to surgical mistakes leading to arterial rupture. 4 grafts were found to be occluded after explant and these samples were excluded from the study. Of the 36 grafts included in the study, 9 samples for each timepoint in C57/BL6 (7 for histological analysis and 2 for open graft SEM) and 3 samples for each timepoint in FVB/N were used.

### Cell tracking

#### Isolation of donor BM-MNCs

Transgenic mice over expressing both firefly luciferase and enhanced green fluorescent protein (eGFP) (FVB-L2G) were purchased from Jackson Laboratories (Bar Harbor, ME, USA). Isolation of BM-MNCs was carried out as previously described [11]. The tibias and femurs from donor FVB-L2G mice (female, 7–8 weeks old, ~ 20 g) were explanted and flushed using a 21-gauged needle with sterile PBS. The bone marrow was then layered on top of Lympholyte^®^-M cell separation media (6 mL per one pair of tibia and femur) and centrifuged at 1250 × g for 25 minutes with deceleration off. The resulting buffy coat was aspirated and washed three times in sterile PBS: 800 × g for 10 mins, 500 × g for 10 mins, and 200 × g for 10 mins. Following the last wash, the pellet of BM-MNCs was resuspended in endothelial basal medium (phenol free) to desired cell concentrations.

#### Bioluminescence imaging

FVB mice (male, 8–9 weeks old, 25 ± 2 g) were purchased from Australian BioResources (Moss Vale, NSW, Australia). Carotid grafting surgery was performed and the animals were allowed to recover before BM-MNCs (1 × 10^6^ cells) were administered via tail vein injection on the same day. D-luciferin was reconstituted at a concentration of 40 mg/ml. To induce bioluminescence, luciferin was given at a total volume of 200 μL via intra-peritoneal injection. Bioluminescence was quantified using the IVIS Series pre-clinical *in vivo* imaging system apparatus (Perkin Elmer). Grafts were explanted at 7, 14 and 28 days post-surgery.

### Histology

Explanted grafts from C57/BL6 mice were fixed overnight in paraformaldehyde (2%) at room temperature. Samples were dehydrated through an ethanol gradient and embedded in paraffin and sectioned at 5 μm transversely from proximal anastomosis to distal anastomosis. Paraffin sections were deparaffinised and rehydrated for haematoxylin and eosin staining and Carstairs staining. For immunohistochemistry staining, sections were deparaffinised and stained with antibodies against SM α-actin (Sigma-Aldrich, a5691, 1:250) and smooth muscle myosin heavy chain 11 (Abcam, ab53219, 1:200) for smooth muscle cells and CD31 (Abcam, ab28364, 1:200) for endothelial cells.

Explanted grafts from FVB-N mice were fixed overnight in paraformaldehyde (2%) at room temperature. Fixed samples were placed in sucrose (30%) for 48 h then embedded in OCT and sectioned at 40 μm. Sections were then stained using standard free-floating techniques with an anti-GFP antibody (Sapphire Bioscience, GTX26662, 1:100).

### Scanning electron microscopy

Electrospun grafts were gold sputter coated (20 nm) and imaged with a JEOL Neoscope Tabletop scanning electron microscopy. For quantification, the widths of 40 fibres were measured within an image for a total of 10 images. Explanted grafts were cut open longitudinally then fixed overnight in paraformaldehyde (2%) at room temperature followed by glutaraldehyde (2.5%) for one hour at room temperature. Samples were then post-fixed with osmium tetraoxide in 0.1 M PB, and dehydrated through an ethanol gradient before drying with hexamethyldisilasane. Samples were gold sputter coated and imaged with a Zeiss Sigma VP FEG scanning electron microscopy.

### Quantitative analysis

Analysis of histological stains was done with ImageJ. Paraffin sections of the graft were selected from 5 points evenly distributed along the graft. From each of these points, 3 slides were selected 5 slides apart and one tissue section from each slide was analysed. Neointima area was quantified represented as a percentage of total lumen area defined by the inner graft wall. SMC content was quantified by measuring the area of SM α-actin using a constant threshold intensity and expressed as a percentage of the neointima area. Similarly, collagen was quantified and expressed as a percentage of the neointima area. Endothelial coverage was quantified by measuring the length of CD31 positive staining along the lumen and expressed as a percentage of the total luminal circumference. GFP+ cell counts were quantified using 10 sections evenly distributed along the graft.

### Statistical analysis

Data are expressed as mean ± SEM and indicated in figures as * p<0.05, ** p<0.01, ***p<0.001, and ****p<0.0001. The data were compared using ANOVA using GraphPad Prism version 6 (Graphpad Software, San Diego, California).

## Results

### Graft characterisation

**Fig 1:**
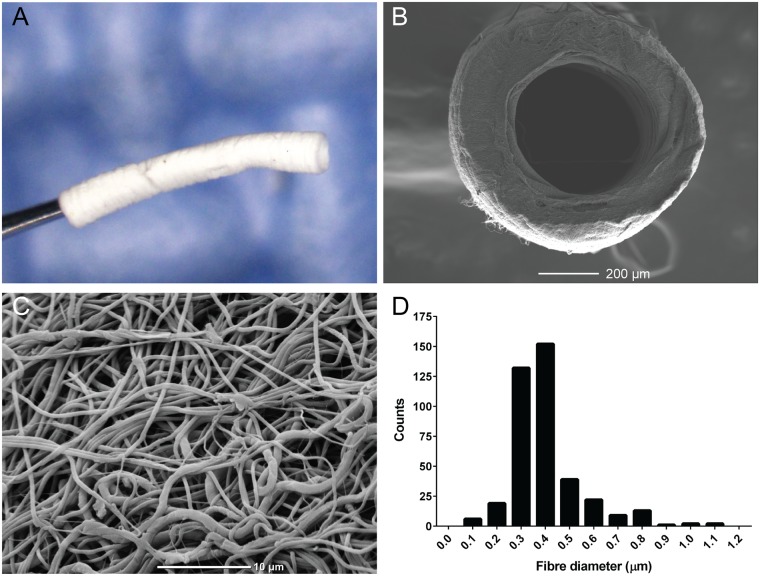
Characterisation of electrospun PCL graft. A: Macroscopic image of PCL graft. B: Scanning electron microscopy images of a transverse cross section. Scale bar = 200 μm C: Scanning electron microscopy images of electrospun fibres on the luminal side. Scale bar = 10 μm. D: Histogram of fibre diameter distribution.

Highly uniform PCL conduits were electrospun with an inner diameter of 500 μm and wall thickness of 100 μm (Fig 1A). Under scanning electron microscopy, the luminal surface and fibrous structure were evident (Fig 1B). At higher magnification, randomly aligned rounded fibres were observed (Fig 1C). Quantification of the fibre characteristics revealed an average fibre diameter of 0.39 ± 0.01 μm, tightly distributed in a range from 0.3‒0.49 μm (Fig 1D).

### Surgical procedure

**Fig 2:**
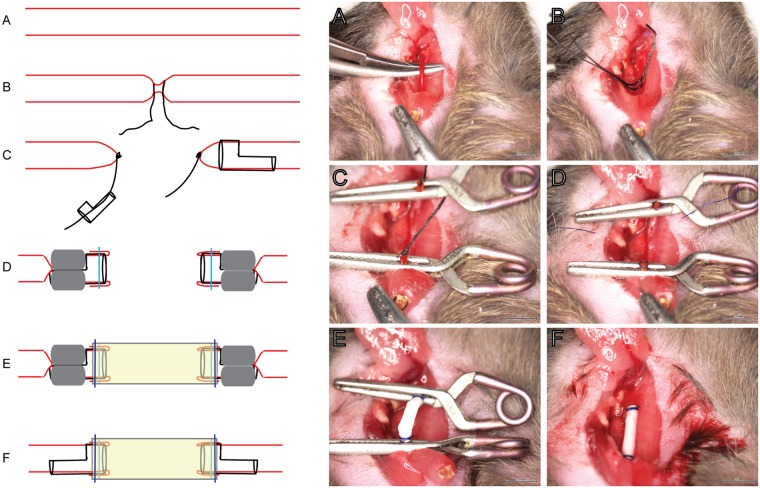
Mouse carotid grafting procedure with corresponding schematic diagram (adapted from [7]). A: Carotid artery was isolated. B: Double ligation at the mid-point. C: Cuffs were placed on each end of the ligation. D, E: Clamps were applied and artery segment was everted over the cuff and fixed in place with suture. F: Graft was secured to each ends of the cuffs. G: Clamps were removed and blood flow confirmed.

The right common carotid artery was mobilised (Fig 2A), care was taken to avoid the vagus nerve and disruption of other microvasculature. Two ligations at the midpoint of the artery were made as close as possible to maximise the length of artery (Fig 2B). After dissecting between the ligations, cuffs were guided onto each end of the artery and clamped with ligated artery protruding (Fig 2C). The overhanging artery was everted and secured on the cuff with a suture (Fig 2D), forming the anastomoses. PCL grafts were sleeved over the everted cuffs and secured (Fig 2E). Clamps were released from the distal side then proximal (Fig 2F).

### Neointimal hyperplasia

#### Area

**Fig 3:**
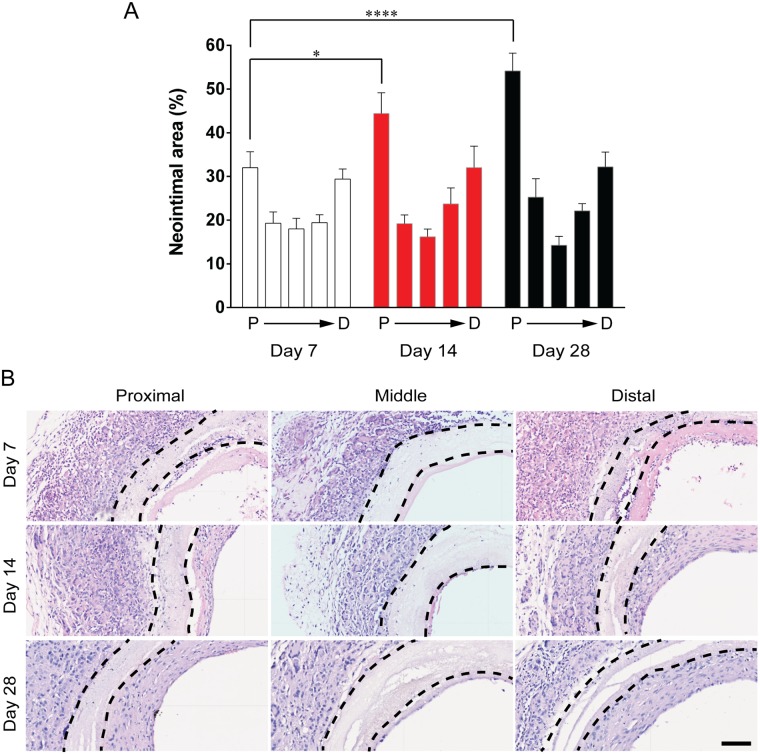
Neointimal area. A: Distribution of NH throughout the graft. Neointimal area represented as a percentage of total luminal area as defined by the inner graft wall. Data expressed as mean ± SEM and analysed using two-way ANOVA, n = 7 animals/timepoint. B: Representative images of H&E staining of cross sections. Black dotted lines indicate the graft wall. Scale bar = 100 μm.

We began by systematically quantifying the neointimal area throughout the grafts at 7, 14 and 28 days. At each time point, we observed a distribution of a developing neointima along the length of the grafts. The neointimal area was greatest at the anastomoses and lowest towards the mid-section (Fig 3A). At 28 days, this effect was most pronounced and the extent of neointimal hyperplasia greatest near the proximal cuff. Neointima cellularity showed a similar trend (S1 Fig) Looking at the temporal increase in hyperplasia at the proximal cuff, neointimal area increased from 32.0 ± 3.7% at day 7 to 44.4 ± 4.7% at day 14 and 54.1 ± 4.0% at day 28 post-surgery (p<0.0001). Representative images demonstrate the progressive increase in hyperplasia area (Fig 3B).

#### Smooth muscle cell content

**Fig 4:**
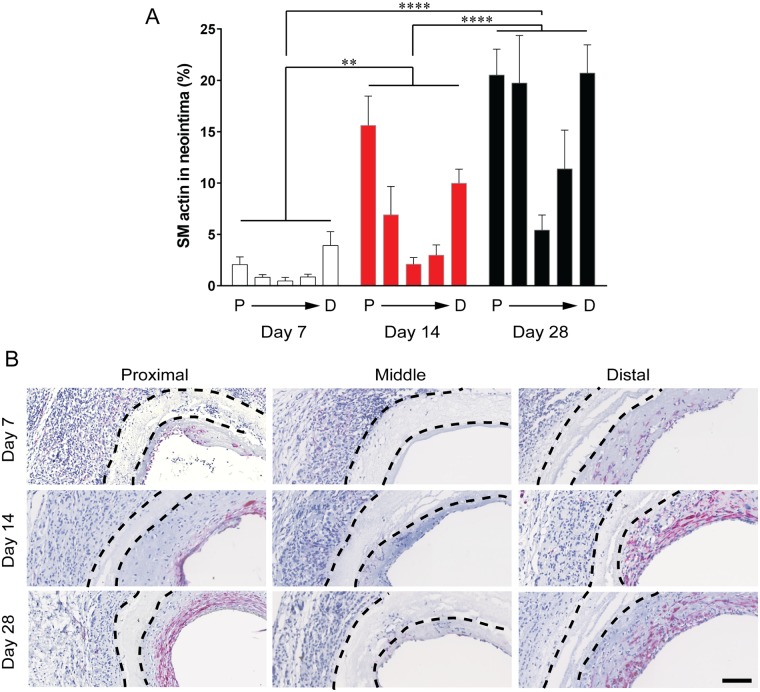
Smooth muscle α-actin content in the neointima. A: Distribution of SM α-actin area throughout the graft. SM α-actin area represented as a percentage of the neointimal area. Data expressed as mean ± SEM and the average SM α-actin content along the length of the graft was analysed using one-way ANOVA, n = 7 animals/timepoint. B: Representative images of cross sections with SM α-actin stained in red, nucleus in blue. Black dotted lines indicate the graft wall. Scale bar = 100 μm.

SMCs are a major component of the neointima. Smooth muscle (SM) α-actin is a marker of SMCs [12]. The distribution of SM α-actin followed the same trends seen for neointimal area, such that there were more SM α-actin+ cells at the anastomoses and less towards the mid-graft (Fig 4A). Also, consistent with the total hyperplasia area, the percentage of average SM α-actin increased significantly from 1.6 ± 0.4% at day 7 to 7.5 ± 1.2% at day 14 post-surgery. SM α-actin+ area continued to increase up to day 28 post-surgery to 15.5 ± 1.7% of the neointimal area. Within the neointima, SM α-actin was observed predominately on the luminal side of the neointima (Fig 4B). Contractile smooth muscle cells (myosin heavy-chain positive) were only evident at day 28 post-surgery, confined to the proximal and distal regions.

#### Collagen content

**Fig 5:**
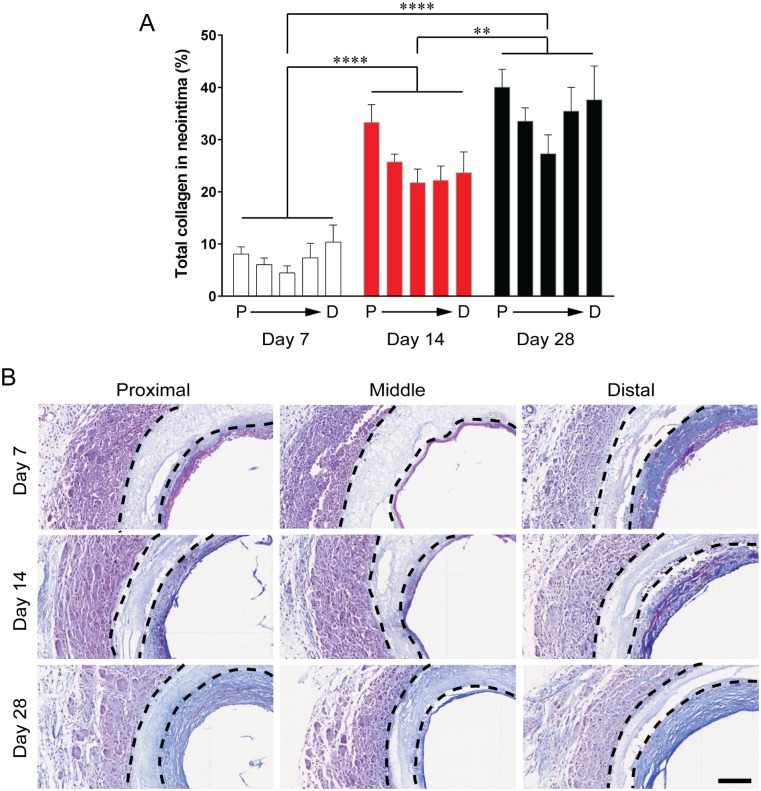
Total collagen content in neointima. A: Distribution of total collagen throughout the graft area represented as a percentage of the neointimal area. Data expressed as mean ± SEM and the average collagen content along the length of the graft was analysed using one-way ANOVA, n = 7 animals/timepoint. B: Representative images of Carstair histological stain of cross sections, collagen in blue, muscle in red. Black dotted lines indicate the graft wall. Scale bar = 100 μm.

Collagen is a major component of newly deposited extracellular matrix (ECM), formed during neointima development. Changes to the collagen distribution throughout grafts within a single timepoint was less varied than the neointima or SM α-actin areas, but most evident at day 28 (Fig 5A). The average total collagen content throughout the graft increased significantly from 7.3 ± 1.0% at day 7 to 25.3 ± 2.1% at day 14 and then 34.8 ± 2.2 at day 28 post-surgery. Representative images showing the distribution of collagen (stained blue) at each time point (Fig 5B).

### Endothelialisation

**Fig 6:**
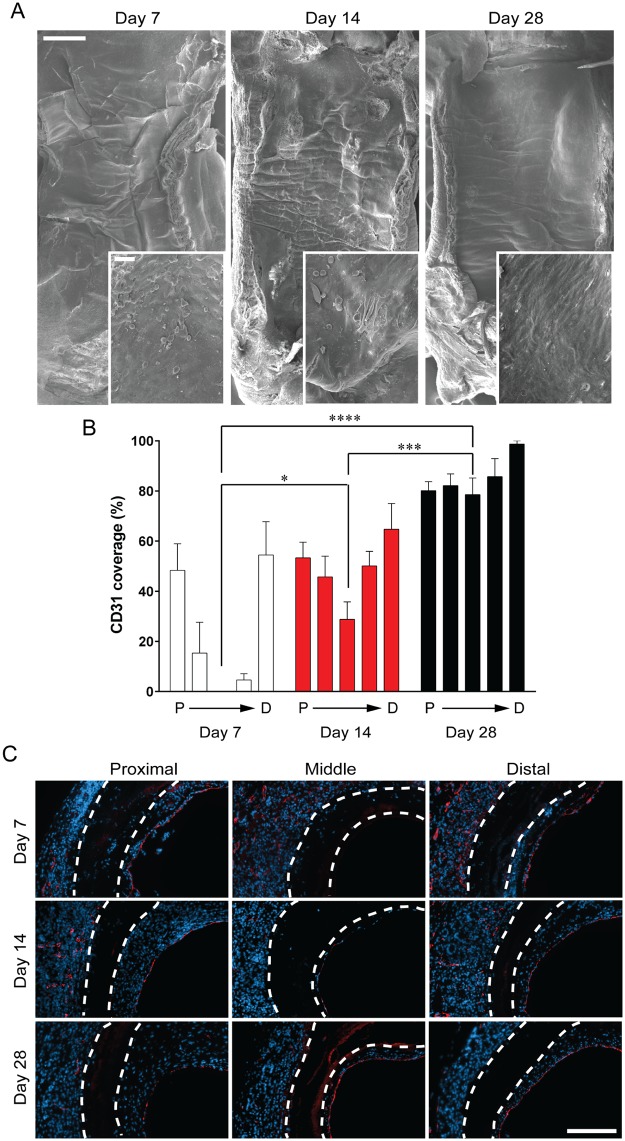
Endothelialisation. A: Scanning electron microscopy images of the luminal side of the graft. Scale bar = 500 μm. Inset scale bar = 50 μm. B: Quantification of CD31 coverage represented as a percentage of total luminal circumference. Data expressed as mean ± SEM and the mid section of the grafts were analysed using one-way ANOVA, n = 7 animals/timepoint. C: Representative images of cross sections with CD31 staining in red, nucleus in blue. White dotted lines indicate the graft wall. Scale bar = 100 μm.

Endothelial cells line arterial walls, providing a non-thrombogenic surface and which also suppresses neointimal hyperplasia. We first examined the cell coverage of implanted grafts at days 7, 14 and 28 post-grafting using SEM (Fig 6A). While the underlying fibrous PCL graft structure is covered at all timepoints, rounded cells and an incomplete surface layer can be seen at days 7 and 14 post-grafting, before a completely uniform cell layer is observed at day 28. To definitively identify and quantify graft endothelialisation, we used CD31 immunohistochemistry. We observed clear differences in endothelial cell distribution throughout the grafts, most evident at day 7, and remaining prominent at day 14 (Fig 6B). At both these timepoints, endothelialisation is greatest at the anastomoses and least in the mid-graft, with no endothelial cells detected in the mid-graft at day 7. By day 28 post-grafting, cell coverage was uniform throughout the grafts. Direct comparison of the endothelialisation of the mid-grafts exemplified the time dependant increase, with no CD31 staining detected at 7 days, partial coverage of 28.8 ± 7.0% at 14 days increasing to 78.5 ± 6.7% by 28 days. Representative images show CD31 positive cells in red (Fig 6C).

### Cell tracking

**Fig 7:**
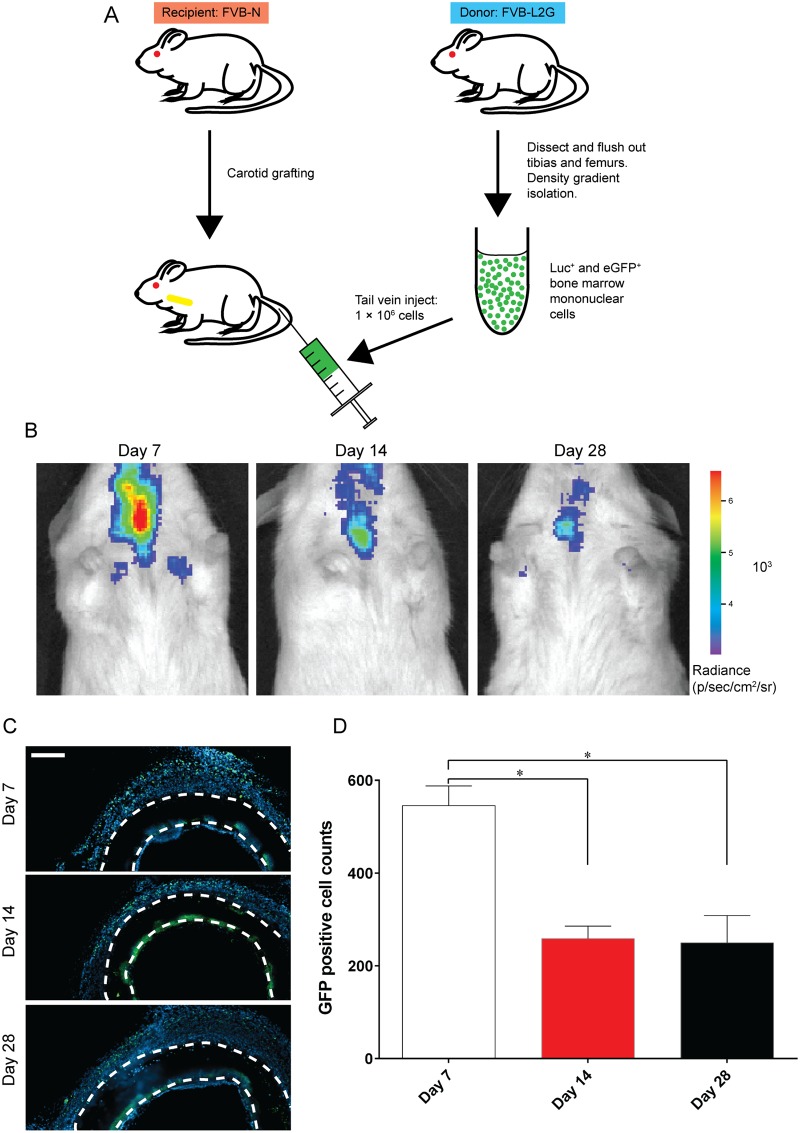
Cell tracking. A: Schematic of experimental design. Bone marrow mono-nuclear cells are isolated from FVB-L2G mice and injected into FVB-N mice that received that carotid grafting. B: Representative IVIS images of carotid grafted mice with BM-MNC injection. Warm colours denote higher signal and cold colours denote lower signal. C: Representative images of cross sections with eGFP staining in green and nucleus in blue. Scale bar = 100 μm. D: Quantification of eGFP positive cells cells. Data expressed as mean ± SEM and analysed using one-way ANOVA, n = 3 animals/timepoint.

Bioluminescent and eGFP^+^ bone marrow mononuclear cells (BM-MNCs) from FVB-L2G mice were injected into FVB-N control recipient mice that had undergone carotid graft surgery (Fig 7A). The temporal and spatial distribution of injected BM-MNCs was tracked non-invasively using IVIS bioluminescent imaging (Fig 7B). Bioluminescence was localised to the carotid area with a more intense signal at 7 days compared to 14 and 28 days. To confirm the presence of injected cells at the graft site, explanted grafts were stained for eGFP. eGFP positive cells were observed at all three time points in the granular tissue surrounding the graft (Fig 7C). Consistent with the bioluminescence signal, quantification of eGFP positive cells showed the highest number of cells at 7 days (545 ± 42), reduced to 258 ± 27 at 14 days, but persistent to 28 days (250 ± 59) (Fig 7D).

## Discussion

In this study, we have comprehensively characterised a mouse model of carotid artery vascular grafting, demonstrating mimicry of clinically important endpoints, including neointimal hyperplasia and endothelialisation. We additionally show the promise of using transgenic mice to bring valuable new tools to the investigation of synthetic graft materials. We chose to validate our model system using electrospun PCL grafts, which have been well characterised previously as rat abdominal aorta replacements [13, 14]. PCL remains a widely used synthetic polymer in tissue engineering because of its controlled degradation, low inflammatory profile and high mechanical strength [13, 15]. The use of cuff anastomoses in the procedure allows for low diameter conduits to be grafted where traditional end to end suture grafting is not feasible. Grafting in the carotid position has implicit advantages over aortic replacement, facilitating the use of non-invasive imaging techniques.

An ideal grafting model should be reflective of the human physiological response to vascular replacement. To characterise this vascular grafting model, we quantified neointima progression and composition and the rate of endothelialisation. In humans, NH initially develops at the anastomoses of small diameter grafts, where the grafted segment joins the native vasculature [16]. This pattern of NH progression has been linked to flow disturbances and compliance mismatch between the native artery and inelastic synthetic materials. Reflecting this pathology, our model showed that NH developed at the anastomoses and decreased towards the mid-section, with the greatest NH at the proximal anastomosis. Clinically, restenosis is defined as 50% occlusion of the lumen of a vessel [17]. In our model, neointimal area gradually increases and reached 53% of the total lumen area by 28 days. SM α-actin and collagen quantification suggest that the hyperplasia is stabilising over time. Compared to other established animal models, such as rat abdominal aorta (3 months) and sheep carotid artery (6 months) [18, 19], the short timeframe of our model is a significant advantage for screening novel synthetic grafts.

The endothelium lining the luminal surface of native vessels actively maintains haemostasis, not only by masking thrombogenic components of the subendothelial connective tissue but also by expressing anti-clotting compounds, including nitric oxide [20]. A complete endothelium is also a critical determinant of vascular lesion formation and areas of injury that rapidly endothelialise have significantly less intimal thickening and restenosis [21]. The clinical performance of synthetic grafts is thus highly dependent on the degree and speed of endothelialisation. Our model shows progressive endothelialisation starting from the anastomoses, evident by day 7. Increases in coverage were observed by 14 days, but the mid-graft was not covered until 28 days. Again, this process occurred in a compressed timeframe, much quicker than the 3 months required for full coverage in rat abdominal aorta grafting [14]. Further, we have identified timepoints where coverage is incomplete (7 and 14 days), allowing for evaluation of synthetic grafts which propose to accelerate endothelialisation. Our model appears to support the ingrowth of endothelium from the anastomotic regions, given the observed mode of progression. However, we recognise that circulating endothelial progenitor cells substantially contribute to prosthesis endothelialisation [22], making them important mediators of implant compatibility. Their contribution could readily be determined in this mouse model using established bone marrow grafting techniques [23]. The mechanism and time course of endothelialisation of our model appear superior to other previous animal models such as canine models which are now seldom used in the field due to spontaneous endothelialisation [5].

Apart from the benefits of low cost and high through put intrinsic to a mouse model, there is also a range of molecular and cellular imaging tools available that have not been fully developed for larger animal models. To begin exemplifying the potential of our model, we injected bioluminescent BM-MNCs derived from FVB-L2G mice and tracked their homing to and engraftment in transplanted PCL grafts. The dual eGFP/luciferase reporters in these cells allow for serial, non-invasive imaging of the cells in live mice, supported by traditional immunohistochemistry analysis. The intense signal observed around the graft at 7 days shows significant BM-MNC homing, while the persistence of bioluminescent at 28 days suggested cell engraftment, validated with positive eGFP staining. This approach has great promise for increasing our understanding the contributions of stem cell containing populations towards tissue regeneration, which while therapeutically promising, have not reached their clinical potential [11]. Importantly, this also provides a valuable tool for the guided development of new synthetic vascular grafts which have enhanced interaction with stem cell therapy.

More broadly, our model enables future evaluation of new synthetic conduits in a range of established disease models relevant to graft failure and prevalent in patients regularly requiring revascularisation of small diameter vessels [1]. Transgenic and knockout mouse strains including those which demonstrate accelerated atherosclerosis (ApoE^-/-^) [24], simulate obesity/diabetes (db/db) [25], or altered eNOS expression [26], have immediate relevance to a more comprehensive evaluation of new graft materials. Mechanistically, the role of inflammatory cells (e.g. macrophages), immune cells (e.g. T cells) and cytokines (e.g. TNFα) in the progression of graft hyperplasia could also be more fully explored [27]. Taken together, grafting of synthetic conduits in a mouse coronary artery will dramatically change the research questions that can be asked in this area, facilitating improved developed of new materials, thereby addressing a long-standing unmet need in vascular surgery.

## Conclusion

In conclusion, we have established a mouse model of vascular grafting that recapitulates clinically relevant features within 28 days. Exemplifying the potential of the model, we showed the non-invasive tracking of BM-MNCs homing and engrafting by utilising a transgenic strain encoded with Luc/GFP dual reporter. We believe this model is a useful tool facilitating the evaluation of novel synthetic grafts.

## Supporting information

S1 Fig Neointima cellularity. Nuclei count of neoinitma using haematoxylin stain. Data expressed as mean ± SEM and the average nuclei count along the length of the graft was analysed using one-way ANOVA, n = 7 animals/timepoint.
(TIF)

S2 Fig Contractile smooth muscle cells in neointima. Representative images of cross section with smooth muscle myosin heavy chain 11 stained in red and nucleus in blue. White dotted lines indicate the graft wall. Scale bar = 100 μm.
(TIF)

## References

[1] FitzGibbon GM, Kafka HP, Leach AJ, Keon WJ, Hooper GD, Burton JR. Coronary bypass graft fate and patient outcome: Angiographic follow-up of 5,065 grafts related to survival and reoperation in 1,388 patients during 25 years. J Am Coll Cardiol. 1996;28: 616–626. 8772748 10.1016/0735-1097(96)00206-9

[2] Veith FJ, Moss CM, Sprayregen S, Montefusco C. Preoperative saphenous venography in arterial reconstructive surgery of the lower extremity. Surgery. 1979;85: 253–256. 424995

[3] Ballyk PD, Walsh C, Butany J, Ojha M. Compliance mismatch may promote graft-artery intimal hyperplasia by altering suture-line stresses. J Biomech. 1998;31: 229–237. 9645537 10.1016/s0197-3975(97)00111-5

[4] Zilla P, Bezuidenhout D, Human P. Prosthetic vascular grafts: Wrong models, wrong questions and no healing. Biomaterials. 2007;28: 5009–5027. 10.1016/j.biomaterials.2007.07.017 17688939

[5] Byrom MJ, Bannon PG, White GH, Ng MKC. Animal models for the assessment of novel vascular conduits. J Vasc Surg. 2010;52: 176–195. 10.1016/j.jvs.2009.10.080 20299181

[6] Lopez-Soler RI, Brennan MP, Goyal A, Wang Y, Fong P, Tellides G, et al Development of a mouse model for evaluation of small diameter vascular grafts. J Surg Res. 2007;139: 1–6. 10.1016/j.jss.2006.07.040 17336332

[7] Zou YP, Dietrich H, Hu YH, Metzler B, Wick G, Xu QB. Mouse model of venous bypass graft arteriosclerosis. Am J Pathol. 1998;153: 1301–1310. 10.1016/S0002-9440(10)65675-1 9777962 PMC1853044

[8] Zhu P, Esckilsen S, Atkinson C, Chen XP, Nadig SN. A simplified cuff technique for abdominal aortic transplantation in mice. J Surg Res. 2016;200: 707–713. 10.1016/j.jss.2015.08.039 26375503 PMC5815517

[9] Ali ZA, Bursill CA, Hu YH, Choudhury RP, Xu QB, Greaves DR, et al Gene transfer of a broad spectrum CC-chemokine inhibitor reduces vein graft atherosclerosis in apolipoprotein E-knockout mice. Circulation. 2005;112: I-235–I-241. 16159823 10.1161/CIRCULATIONAHA.104.526129

[10] Ali ZA, Alp NJ, Lupton H, Arnold N, Bannister T, Hu Y, et al Increased in-stent stenosis in ApoE knockout mice—Insights from a novel mouse model of balloon angioplasty and stenting. Arteriosclerosis Thrombosis and Vascular Biology. 2007;27: 833–840. 10.1161/01.ATV.0000257135.39571.5b 17204666

[11] Sheikh AY, Huber BC, Narsinh KH, Spin JM, van der Bogt K, de Almeida PE, et al In vivo functional and transcriptional profiling of bone marrow stem cells after transplantation into ischemic myocardium. Arterioscler Thromb Vasc Biol. 2012;32: 92–102. 10.1161/ATVBAHA.111.238618 22034515 PMC3241895

[12] Rensen SSM, Doevendans P, van Eys G. Regulation and characteristics of vascular smooth muscle cell phenotypic diversity. Neth Heart J. 2007;15: 100–108. 17612668 10.1007/BF03085963 PMC1847757

[13] Nottelet B, Pektok E, Mandracchia D, Tille JC, Walpoth B, Gurny R, et al Factorial design optimization and in vivo feasibility of poly(epsilon-caprolactone)-micro- and narofiber-based small diameter vascular grafts. Journal of Biomedical Materials Research Part A. 2009;89A: 865–875. 10.1002/jbm.a.32023 18465817

[14] de Valence S, Tille JC, Mugnai D, Mrowczynski W, Gurny R, Moller M, et al Long term performance of polycaprolactone vascular grafts in a rat abdominal aorta replacement model. Biomaterials. 2012;33: 38–47. 10.1016/j.biomaterials.2011.09.024 21940044

[15] Williamson MR, Black R, Kielty C. PCL–PU composite vascular scaffold production for vascular tissue engineering: Attachment, proliferation and bioactivity of human vascular endothelial cells. Biomaterials. 2006;27: 3608–3616. 10.1016/j.biomaterials.2006.02.025 16530824

[16] Bassiouny HS, White S, Glagov S, Choi E, Giddens DP, Zarins CK. Anastomotic intimal hyperplasia: mechanical injury or flow induced. J Vasc Surg. 1992;15: 708–717. 1560562 10.1067/mva.1992.33849

[17] Mitra AK, Agrawal DK. In stent restenosis: bane of the stent era. J Clin Pathol. 2006;59: 232–239. 10.1136/jcp.2005.025742 16505271 PMC1860348

[18] Pektok E, Nottelet B, Tille JC, Gurny R, Kalangos A, Moeller M, et al Degradation and Healing Characteristics of Small-Diameter Poly(epsilon-Caprolactone) Vascular Grafts in the Rat Systemic Arterial Circulation. Circulation. 2008;118: 2563–2570. 10.1161/CIRCULATIONAHA.108.795732 19029464

[19] Soldani G, Losi P, Bernabei M, Burchielli S, Chiappino D, Kull S, et al Long term performance of small-diameter vascular grafts made of a poly(ether)urethane-polydimethylsiloxane semi-interpenetrating polymeric network. Biomaterials. 2010;31: 2592–2605. 10.1016/j.biomaterials.2009.12.017 20035992

[20] Xue L, Greisler HP. Blood vessels In: Lanza R, Langer R, Vacanti J, editors. Principles of tissue engineering. 2nd ed San Diego: Academic Press; 2000 pp. 427–445.

[21] Lau WC, Waskell LA, Watkins PB, Neer CJ, Horowitz K, Hopp AS, et al Atorvastatin reduces the ability of clopidogrel to inhibit platelet aggregation: a new drug-drug interaction. Circulation. 2003;107: 32–37. 12515739 10.1161/01.cir.0000047060.60595.cc

[22] Shi Q, Rafii S, Wu MH, Wijelath ES, Yu C, Ishida A, et al Evidence for circulating bone marrow-derived endothelial cells. Blood. 1998;92: 362–367. 9657732

[23] Asahara T, Masuda H, Takahashi T, Kalka C, Pastore C, Silver M, et al Bone marrow origin of endothelial progenitor cells responsible for postnatal vasculogenesis in physiological and pathological neovascularization. Circ Res. 1999;85: 221–228. 10436164 10.1161/01.res.85.3.221

[24] Zhang SH, Reddick RL, Piedrahita JA, Maeda N. Spontaneous hypercholesterolemia and arterial lesions in mice lacking apolipoprotein E. Science. 1992;258: 468–471. 1411543 10.1126/science.1411543

[25] Kobayashi K, Forte TM, Taniguchi S, Ishida BY, Oka K, Chan L. The db/db mouse, a model for diabetic dyslipidemia: molecular characterization and effects of Western diet feeding. Metabolism. 2000;49: 22–31. 10647060 10.1016/s0026-0495(00)90588-2

[26] Champion HC, Bivalacqua TJ, Greenberg SS, Giles TD, Hyman AL, Kadowitz PJ. Adenoviral gene transfer of endothelial nitric-oxide synthase (eNOS) partially restores normal pulmonary arterial pressure in eNOS-deficient mice. Proc Natl Acad Sci U S A. 2002;99: 13248–13253. 10.1073/pnas.182225899 12237402 PMC130619

[27] Hui DY. Intimal hyperplasia in murine models. Curr Drug Targets. 2008;9: 251–260. 18336244 10.2174/138945008783755601 PMC2829189

