# Supplementary figures and images for: Evaluation of synthetic vascular grafts in a mouse carotid grafting model
Source: PLoS One. 2017 Mar 29;12(3):e0174773. doi: 10.1371/journal.pone.0174773 (PMC5371373; doi:10.1371/journal.pone.0174773)

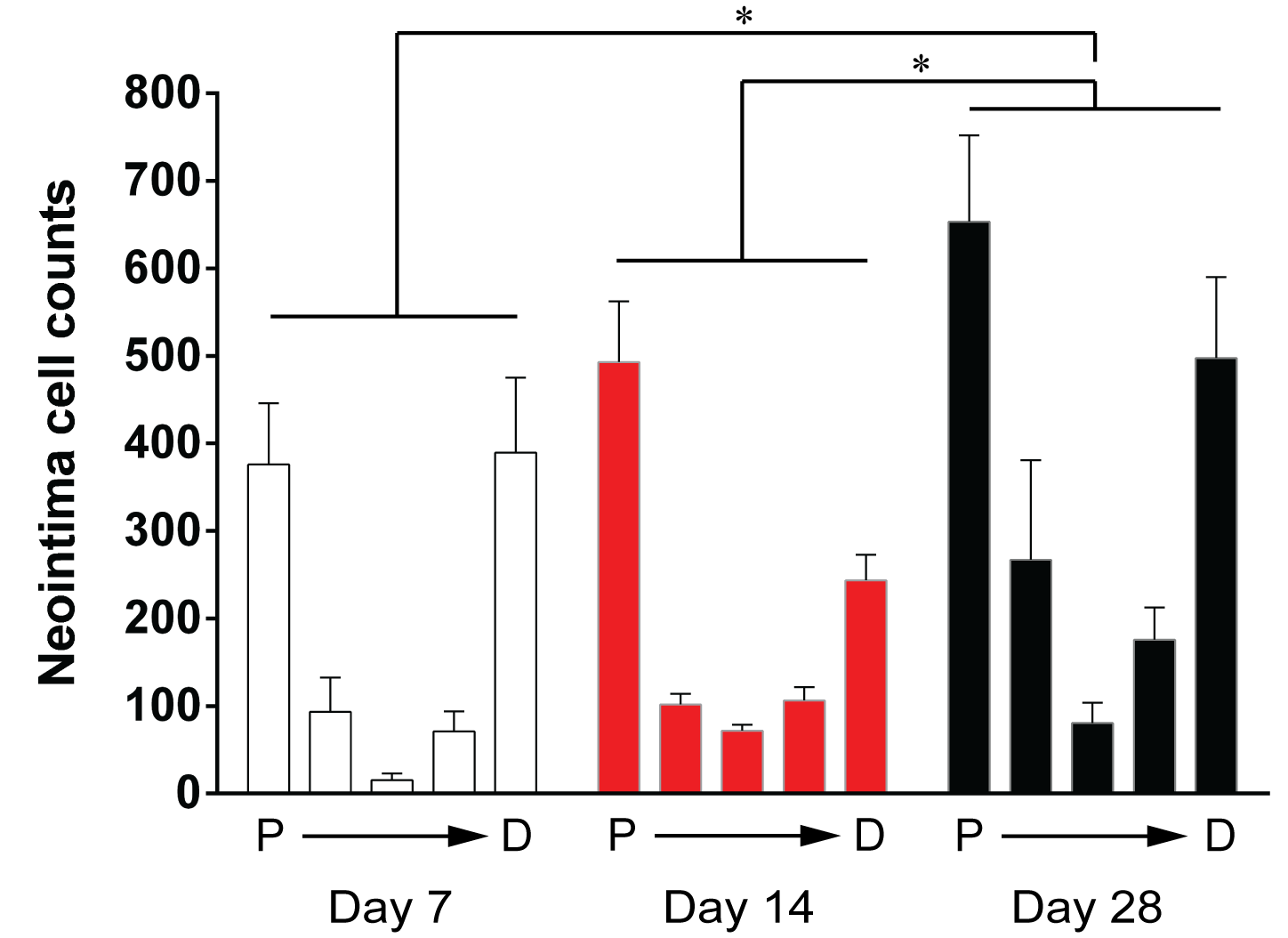

Supplement: S1 Fig — Nuclei count of neoinitma using haematoxylin stain. Data expressed as mean ± SEM and the average nuclei count along the length of the graft was analysed using one-way ANOVA, n = 7 animals/timepoint. (TIF) [file pone.0174773.s001.tif]

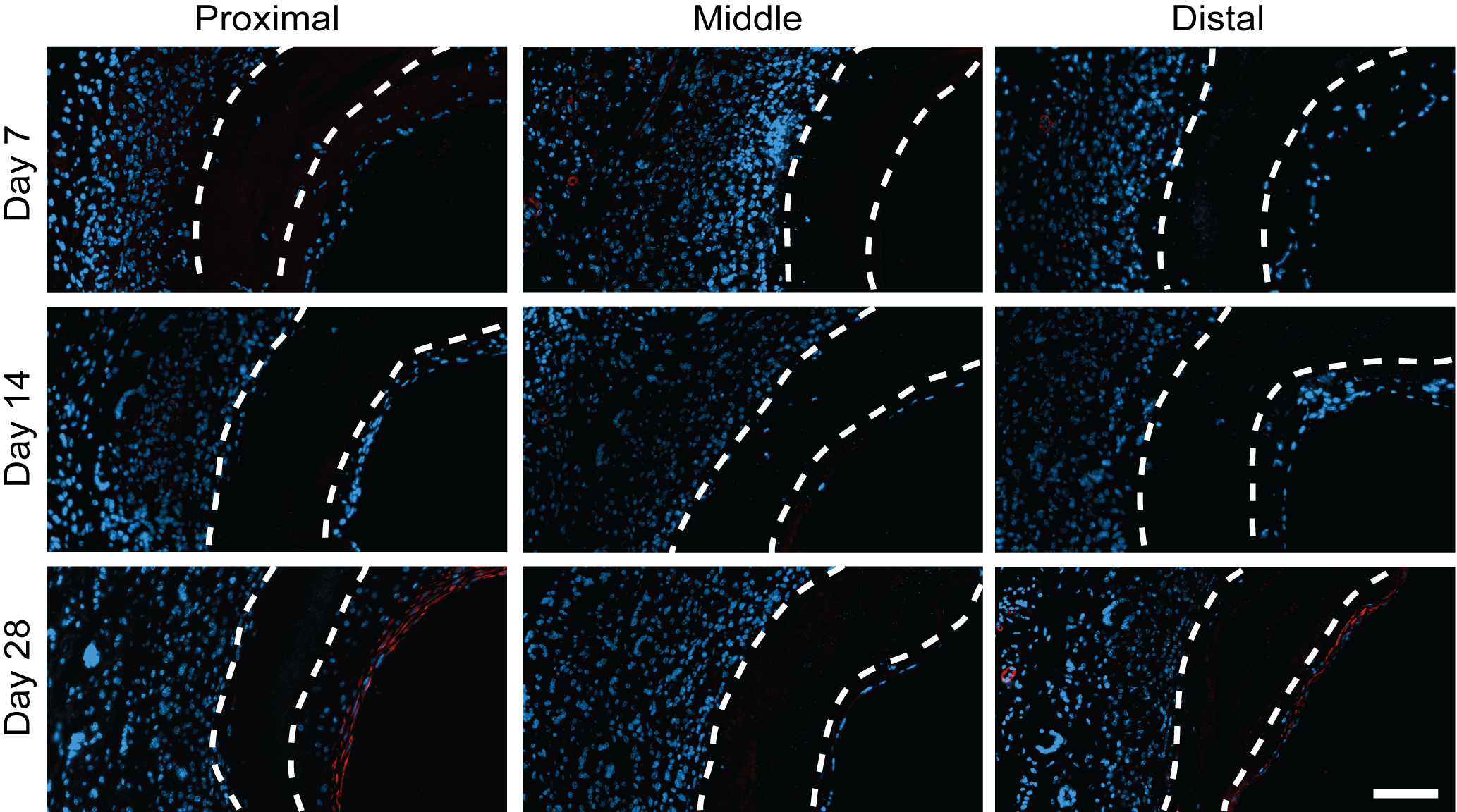

Supplement: S2 Fig — Representative images of cross section with smooth muscle myosin heavy chain 11 stained in red and nucleus in blue. White dotted lines indicate the graft wall. Scale bar = 100 μm. (TIF) [file pone.0174773.s002.tif]
